# Repurposing of Some Natural Product Isolates as SARS-COV-2 Main Protease Inhibitors via In Vitro Cell Free and Cell-Based Antiviral Assessments and Molecular Modeling Approaches

**DOI:** 10.3390/ph14030213

**Published:** 2021-03-04

**Authors:** Hossam M. Abdallah, Ali M. El-Halawany, Alaa Sirwi, Amr M. El-Araby, Gamal A. Mohamed, Sabrin R. M. Ibrahim, Abdulrahman E. Koshak, Hani Z. Asfour, Zuhier A. Awan, Mahmoud A. Elfaky

**Affiliations:** 1 Department of Natural Products and Alternative Medicine, Faculty of Pharmacy, King Abdulaziz University, Jeddah 21589, Saudi Arabia; asirwi@kau.edu.sa (A.S.); gahussein@kau.edu.sa (G.A.M.); aekoshak@kau.edu.sa (A.E.K.); melfaky@kau.edu.sa (M.A.E.); 2 Department of Pharmacognosy, Faculty of Pharmacy, Cairo University, Cairo 11562, Egypt; ali.elhalawany@pharma.cu.edu.eg; 3 Department of Pharmaceutical Chemistry, Faculty of Pharmacy, Ain Shams Universit, Cairo 11566, Egypt; amr.m.amin@pharma.asu.edu.eg; 4 Department of Pharmacognosy, Faculty of Pharmacy, Al-Azhar University, Assiut Branch, Assiut 71524, Egypt; 5 Batterjee Medical College, P.O. Box 6231, North Obhur, Prince Abdullah Al-Faisal Street, Jeddah 21442, Saudi Arabia; sabrinshaur@gmail.com; 6 Department of Pharmacognosy, Faculty of Pharmacy, Assiut University, Assiut 71526, Egypt; 7 Department of Medical Microbiology and Parasitology, Faculty of Medicine, King Abdulaziz University, Jeddah 21589, Saudi Arabia; hasfour@hotmail.com; 8 Department of Clinical Biochemistry, Faculty of Medicine, King Abdulaziz University, Jeddah 21589, Saudi Arabia; zawan@kau.edu.sa

**Keywords:** SARS-CoV-2 main protease, coronavirus, virtual screening, acetoside, naringenin, apigenin-7-*O*-glucoside, sennoside B, pharmacophore, SARS-CoV-2 Egyptian strain

## Abstract

The emergence of the SARS-CoV-2 pandemic has prompted scientists to search for an efficient antiviral medicine to overcome the rapid spread and the marked increase in the number of patients worldwide. In this regard natural products could be a potential source of substances active against coronavirus infections. A systematic computer-aided virtual screening approach was carried out using commercially available natural products found on the Zinc Database in addition to an in-house compound library to identify potential natural product inhibitors of SARS-CoV-2 main protease (M^PRO^). The top eighteen hits from the screening were selected for in vitro evaluation on the viral protease (SARS-CoV-2 M^PRO^). Five compounds (naringenin, 2,3′,4,5′,6-pentahydroxybenzophenone, apigenin-7-*O*-glucoside, sennoside B, and acetoside) displayed high activity against the viral protein. Acteoside showed similar activity to the positive control GC376. The most potent compounds were tested in vitro on SARS-CoV-2 Egyptian strain where only naringenin showed moderate anti-SARS-CoV-2 activity at non-cytotoxic micromolar concentrations in vitro with a significant selectivity index (CC_50_/IC_50_ = 178.748/28.347 = 6.3). Moreover; a common feature pharmacophore model was generated to explain the requirements for enzyme inhibition by this diverse group of active ligands. These results pave a path for future repurposing and development of natural products to aid in the battle against COVID-19.

## 1. Introduction

In December 2019 a new coronavirus gave rise to an outbreak of pulmonary disease in Wuhan City (Hubei Province, China) and since then it has disseminated globally [1,2]. It has been called SARS-CoV-2 [3] because the RNA genome is ≈82% analogous to the SARS-CoV (SARS coronavirus); both viruses belong to clade b of the *Betacoronavirus* genus. The disease produced by SARS-CoV-2 is named COVID-19. The cases were linked to the Huanan animal and seafood market in Wuhan at the beginning of the outbreak, and an effective human-to-human transition resulted in a more rapid increase in the number of cases [1,2]. The genome of SARS-CoV consists of 29,700 nucleotides and its replicase gene involves 21,000 nucleotides, therefore it is bigger than a typical picornavirus whole genome. The replicase encodes two overlapping polyproteins, pp1ab (790 kDa) and pp1a (486 kDa) that induce all the processes required for viral transcription and replication in CoVs [4]. From each polyprotein, functional polypeptides are liberated through comprehensive proteolytic process by the 33.8-kDa Mpro (main protease) (also named the 3C-like protease, 3CLpro). The Mpro significance in the life cycle of virus makes it an effective target for discovering drugs directed towards various CoV infections [5].

Due to the marked rise in the number of patients and the speedy spread of this disease worldwide, an efficacious antiviral therapeutic is urgently needed. The scientific community has begun an extensive effort to expedite the development of new drugs with antiviral potential to lessen COVID-19 fatalities [6]. Natural products are a prosperous reservoir of bioactive molecules with antiviral capacity, and thus could have advantages as remarkable therapeutic agents against CoV infections [7,8]. Some natural metabolites and their derivatives, in addition to traditional medicine products, were known to have SARS-CoV-2 inhibitory potential [9]. Furthermore, some medicinal plants ameliorated the health condition of patients having severe or mild symptoms and stopped healthy persons from becoming infected with SARS-CoV-2 [10].

In silico studies on thymohydroquinone, nigelledine, hederagenin, α-hederin, and thymoquinone from *Nigella sativa* revealed that these compounds potentially inhibited the replication of SARS-CoV-2 and bond to host cell receptors [11]. Just recently, Lung et al. reported that theaflavin, a biflavonoid from black tea, had prominent docking affinities in the SARS-CoV-2 RNA-dependent RNA polymerase catalytic site using in silico approaches [12]. An ethanol extract of *Scutellaria baicalensis* was reported to block the activity of the Mpro of SARS-CoV-2, 3CLpro, and SARS-CoV-2 replication in vitro [13]. Furthermore, its major constituent, baicalein, was found to strongly inhibit SARS-CoV-2 3CLpro activity and viral replication by docking to the substrate-binding pocket of SARS-CoV-2 3CLpro through interaction with two catalytic residues (the oxyanion loop and the crucial S1/S2 sub-sites) thus preventing the peptide substrate from reaching the active site [14]. Quimque et al. demonstrated that the terpenoid 11α-dehydroxyisoterreulactone A, fumiquinazoline alkaloids norquinadoline A, scedapin C, andquinadoline B, and the polyketide isochaetochromin D1, possessed high binding capacities to the target proteins: 3CLpro, PLpro (papain-like protease), nsp15 (non-structural protein 15), RdRp (RNA-directed RNA polymerase), and the spike binding domain to GRP78 [15].

In the current study, a systematic computer-aided virtual screening approach was carried out to identify potential natural product inhibitors of the novel virus SARS-CoV-2 main protease (M^PRO^). Our approach was initiated by conducting a high throughput virtual screening (HTVS) campaign of commercially available natural products found on the Zinc database [16], in addition to an in-house compound library. The top 18 hits of the screening were selected for in vitro evaluation, as inhibitors, on the viral protease (SARS-CoV-2 M^PRO^), whereby five compounds were found to be potentially active. Following in vitro studies, more detailed covalent/non-covalent docking experiments were carried out to investigate the mechanism of inhibition of the identified inhibitors. Moreover; a pharmacophore model for the active hits was generated using the active hits and the co-crystallized ligand.

## 2. Results and Discussion

### 2.1. High-Throughput Virtual Screening of Compound Databases

The SARS-CoV-2 M^PRO^ is a 306 amino acid non-structural protein also recognized as nsp5. The protease parts itself between nsp4 and nsp6 where the monomer is produced [17]. A homodimerization process takes place where an N-terminal finger from each monomer interact together to stabilize the dimer [18]. The monomer itself is essentially inactive, where the activity is initiated upon dimer formation [19]. Following dimerization, M^PRO^ then processes the remainder of the viral polypeptide with sequence-specific sites to activate other non-structural proteins [20].

SARS-CoV-2 M^PRO^ is formed of three domains with high homology with its analogues in SARS-CoV and MERS-CoV. Domains I and II consisted of β-barrels with an antiparallel arrangement. Domain II is linked to domain III via a loop-comprising residue 185–200. The substrate binding site is found between domains I and II where a catalytic diad is present [17]. This diad is composed of His41 and Cys145. The cleavage mechanism is a stepwise process commenced by proton abstraction from the thiol group of Cys145 via the histidine ring of His41. The thiolate anion attacks the target peptide bond cleaving the substrate where the products are released via N-protonation and thioester hydrolysis of the N-terminal and C-terminal, respectively [21].

The aim of current investigation is to characterize potential SARS-CoV-2 M^PRO^ inhibitors from commercially available natural product databases. To pursue this goal, we conducted a rational molecular modeling and screening approach of natural products found in the Zinc database and in-house compounds. The screening comprised non-covalent molecular docking of 8793 natural compounds into SARS-CoV-2 M^PRO^ active site (PDB entry: 6w63). This crystal structure was chosen due to the presence of a non-covalent inhibitor co-crystallized with the protein. All the ligands were docked using standard precision for rapid screening combined with maximum accuracy.

The docking protocol was validated via redocking of the co-crystallized inhibitor where an acceptable RMSD value of 1.56 Å was calculated. Visual inspection of the re-docked pose of the co-crystallized inhibitor showed that they had almost identical binding poses (Figure S1). The re-docked pose revealed the same interactions with the active site as the native pose with an extra hydrogen bond with the backbone carbonyl oxygen of Thr26 due to in-place flipping of the imidazole ring of the inhibitor. This run shows that the docking protocol is valid and reliable for the prediction of possible inhibitors in our compound database. Docking of the natural product database yielded 18 in silico hits that were selected for further in vitro enzyme inhibitory assay (Table 1 and Table S1).

### 2.2. Results of In-Vitro Inhibition of SARS-CoV-2 M^PRO^ by Selected Natural Product Isolates

**Figure 1:**
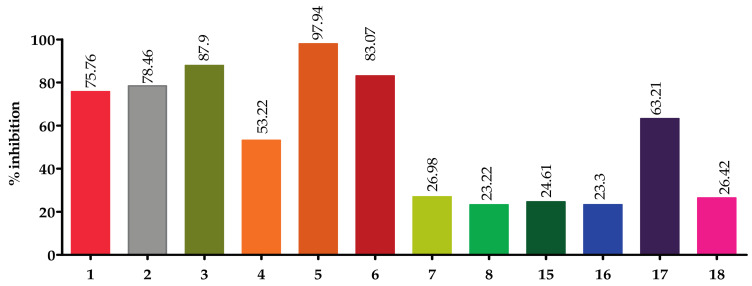
Screening of top hit compounds towards SARS-CoV-2 M^PRO^ utilizing FRET assay.

Screening of the top hit compounds towards SARS-CoV-2 M^PRO^ was performed utilizing the FRET assay and the known inhibitor GC376 as positive control. The results revealed that naringenin, 2,3′,4,5′,6-pentahydroxybenzophenone, apigenin-7-*O*-glucoside, sennoside B, and acteoside are the most potent inhibitors, with ≥75% inhibition of enzyme activity at 100 µM concentration (97.94%, 87.9%, 83.07%, 78.46%, and 75.76%, respectively) (Figure 1). On the other hand, ochnaflavonone-4′-methyl ether and thymoquinone showed moderate activities with 53% and 63% inhibition, respectively, at 100 µM concentration.

**Figure 2:**
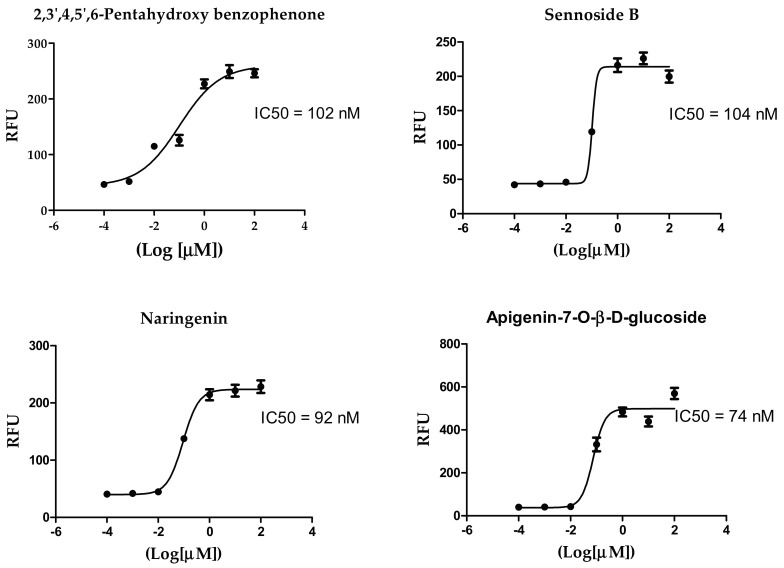

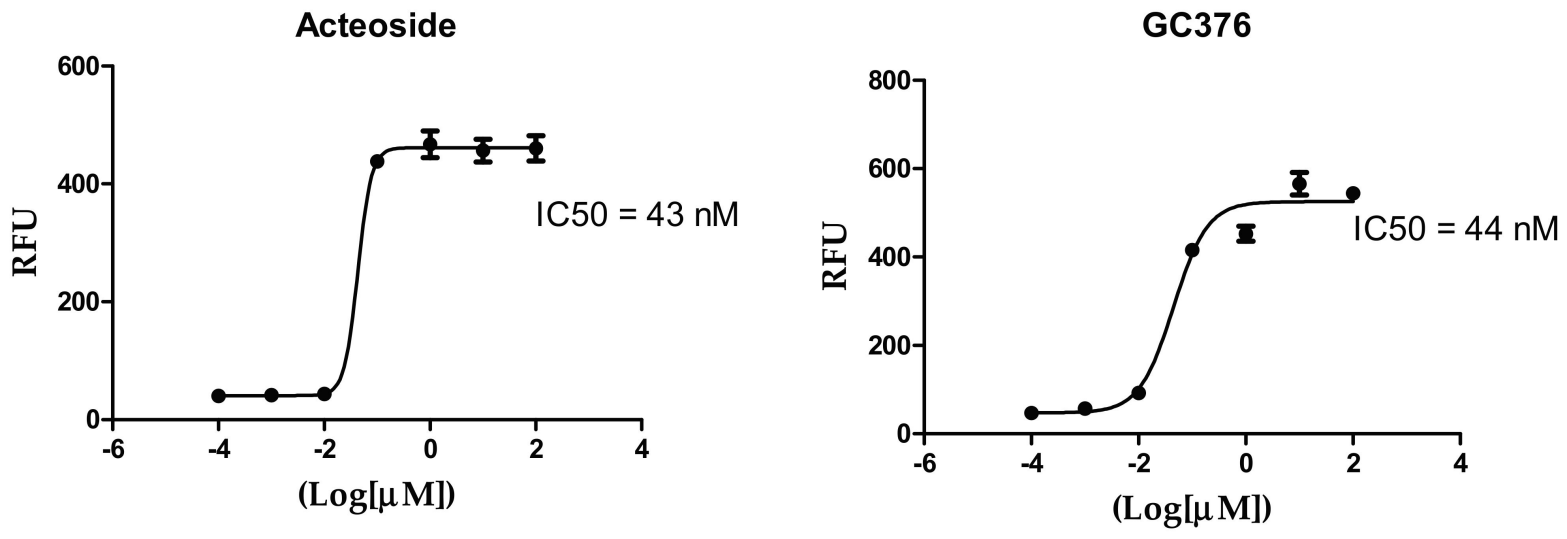
Inhibition of 3CL Protease enzyme activity by compounds; 2,3′,4,5′,6-Pentahydroxybenzophenone, sennoside B, narigenin, apigenin-7-O-β-d-glucoside, and acteoside.

The IC_50_ values of the most potent inhibitors were 102, 104, 92, 74 and 43 nM, respectively, compared to 44 nM of the positive control (Table 2, Figure 2). The results of both docking study and in vitro enzyme inhibition assay were in good agreement in most cases with some exceptions as follows; the most active compounds in docking study showed the most potent enzyme inhibition effects except in case of ochnaflavonone-4′-methyl ether which showed lower activity despite its promising docking score. On the other hand, all the compounds with docking score lower than that of apigenin-7-O-B-D glucoside did not show any significant enzyme inhibitory effect except for thymoquinone which showed moderate activity.

### 2.3. Pose Prediction of the In Vitro-Active Compounds

In vitro compound screening showed that our top 18 hits yielded five compounds that were significantly active in vitro against SARS-CoV-2 M^PRO^. To understand the mechanism of binding of these active compounds, docking experiments were conducted once more. This time, extra precision docking was performed to obtain the most accurate docking pose for each inhibition [22]. Except for naringenin, docking results were almost perfectly aligned with in vitro results, where the docking scores matched the practically measured IC_50_ values. In the in vitro screening, naringenin came third but it was the last in the docking experiment.

**Figure 3:**
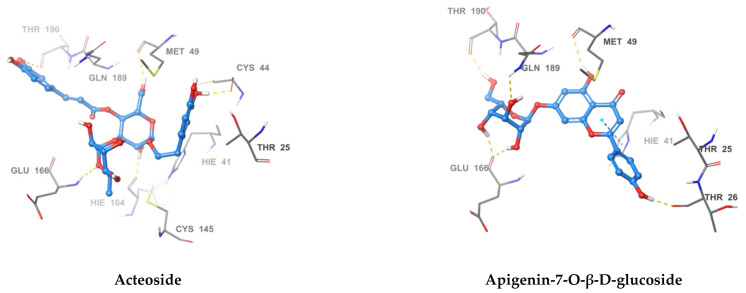

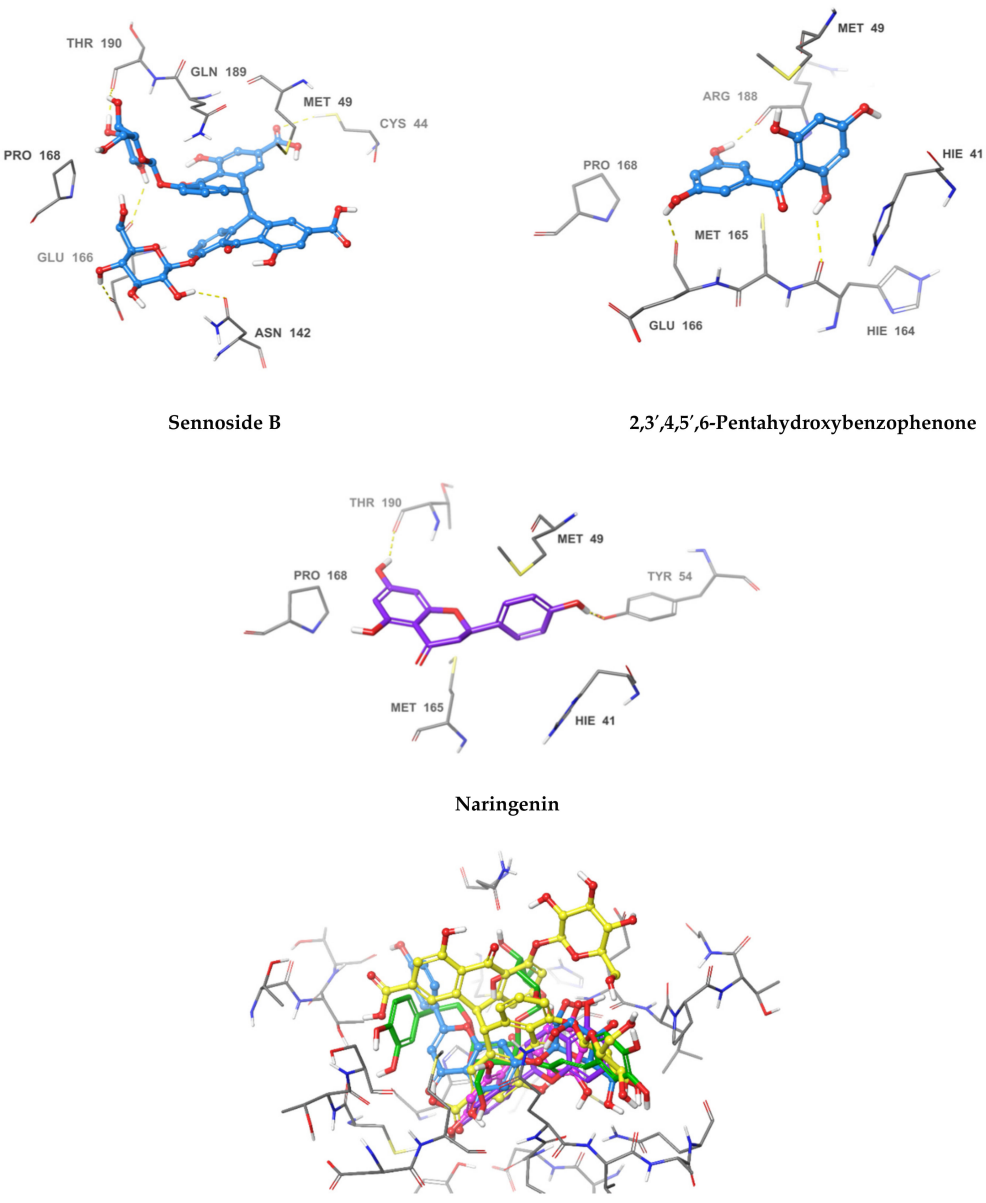
3D Interaction diagrams of acteoside, apigenin-7-O-β-d-glucoside, sennoside B, 2,3′,4,5′,6-pentahydroxy benzophenone, and naringenin, respectively. The final image shows an overlay of the docked compounds in the active site of SARS-CoV-2 M^PRO^. The compounds are colored in green, blue, yellow, magenta and violet respectively.

Docked poses showed that the inhibitors were able to form a network of hydrogen bonds that anchored them to the binding site of the enzyme. Comparison of the binding pose of the studied inhibitors and the co-crystallized ligand revealed that they performed many common interactions, while compounds under investigation showed some extra interactions with polar residues. Table 3 summarizes the interactions between each of the active compounds and the active site. 3D and 2D interaction diagrams of the inhibitors in the active site of SARS-CoV-2 M^PRO^ can be found in the (Figure 3 and Figure S3). The positive control, GC376, is shown to inhibit SARS-CoV-2 M^PRO^ via a covalent mechanism [23].

The most active compound, acteoside (compound **1**), was able to form a hydrogen bond with the backbone amide nitrogen of Glu166, similar to the co-crystallized ligand. A hydrogen bond is noted between acteoside and the side chain carbonyl of Asn142, in a very near position to the hydrogen bond between the co-crystallized ligand and the nitrogen of Gly143. Although acteoside lacks the pi-pi interaction formed between the co-crystallized ligand and His41 and the hydrogen bond with His163, it forms four extra hydrogen bonds with Cys44, Met49, His164, and Thr190. Apigenin-7-*O*-B-d-glucoside (compound **6**) has two hydrogen bonds in common with acteoside with Glu166 and Thr190. However, **2** shows hydrogen bonding with three other residues, the carbonyl of Thr26 and Met49 and the side chain nitrogen of Gln189.

Similar to compounds **1** and **6**, sennoside B (compound **2**) forms hydrogen bonds with Glu166 and Thr190. Two hydrogen bonds are noted with the carbonyl of Thr190 and two other hydrogen bonds with Thr190, one with the carboxylic acid side chain and one with the backbone carbonyl. Two extra hydrogen bonds are noted with the side chain carbonyl of Asn142 and the thiol group of Cys44. 2,3′,4,5′,6-Pentahydroxybenzophenone (compound **3**) was able to hydrogen bond to the carbonyl of Glu166 with the formation of three additional hydrogen bonds with the carbonyls of Hie164 and Arg188 and the phenolic hydroxyl group of Tyr54. Finally, naringenin (compound **5**) was able to form hydrogen bonds with the carbonyl of Thr190 and the phenolic group of Tyr54.

In comparison with the covalent SARS-CoV-2 M^PRO^ GC376, it is noted that all the compounds except for naringenin, shared hydrogen bonding interactions with Glu166. The positive control also forms a hydrogen bond to Gln189 similar to apigenin-7-*O*-β-d-glucoside. GC376 forms extra hydrogen bonds with Gly143 and His163 not found in any of our compounds. The presence of hydrogen bonding interactions between our compounds and either Glu166 and Thr190 or both highlights the importance of interaction with these residues for inhibition. The hydrogen bond with Gln166 seems to be of superior importance as it is a shared feature with the positive control.

### 2.4. Acteoside Binds to SARS-CoV-2 M^PRO^ in a Non-Covalent Manner

**Figure 4:**
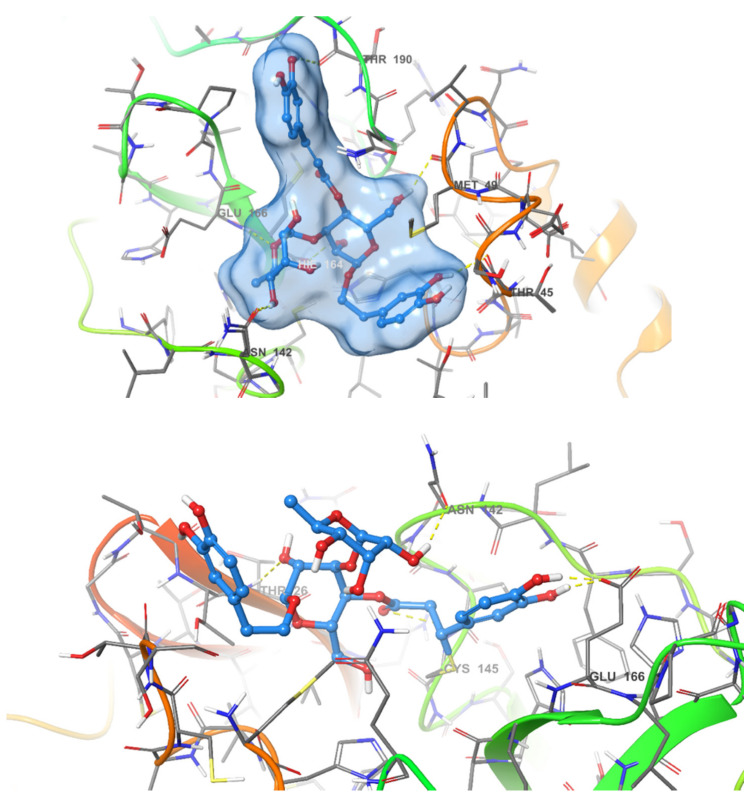
Covalent (**bottom**) and non-covalent (**top**) docking poses of acteoside in the active site of SARS-CoV-2 M^PRO.^

A very intriguing aspect of the most active compound, acteoside, was the presence of an α,β-unsaturated ester which could act as a Michael acceptor and form a covalent bond with the enzyme. Covalent inhibitors of SARS-CoV-2 M^PRO^ have been frequently reported in literature [18,24]. These inhibitors form a covalent bond with the thiol group of Cys145. This feature of acteoside inspired us to investigate whether this compound inhibits the enzyme in a covalent or non-covalent manner. Other compounds in our active series do not contain reactive groups which could react with Cys145. Apigenin-7-*O*-β-d-glucoside, despite containing a cyclic α-β unsaturated ketone, was not accepted by CovDock as a Michael acceptor for covalent docking. To answer this question regarding acteoside, we carried out covalent docking experiments on acteoside using the PDB structure 6Y2F. This crystal structure was used due to the presence of a co-crystallized covalent inhibitor bound to the protease. Docking was performed using a pose-prediction mode for maximum accuracy. An extra energy minimization step was performed for both covalent and non-covalent poses of acteoside and the free energy difference (∆G) was calculated for both poses according to the following equation:

$$\Delta G=G_{\mathrm{Complex}}-\left(G_{\mathrm{Protein}}+{\;G}_{\mathrm{Ligand}}\right)$$

where ∆G for the covalent and non-covalent binding poses were −8.978 and −91.494 kcal/mol respectively. These energy values conclude that the potential binding mode of acteoside to the protease is non-covalent in nature (Figure 4).

### 2.5. Antiviral Activity of the Most Potent SARS COV-2 Viral Main Protease Inhibitors

**Figure 5:**
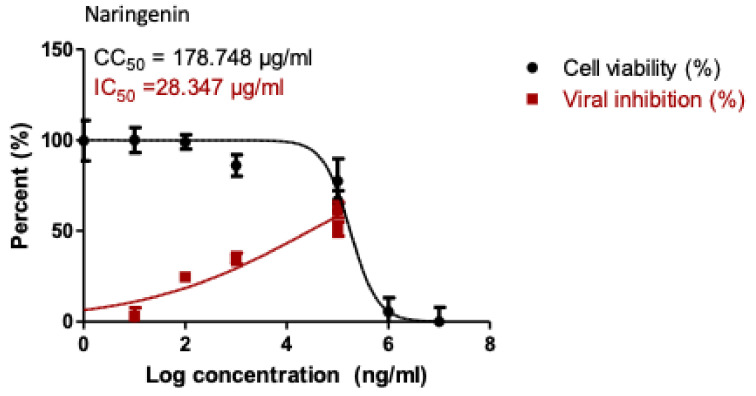
Dose-inhibition curves for naringenin. Inhibitory concentration 50% (IC50) values were calculated using nonlinear regression analysis of GraphPad Prism software (version 5.01) by plotting log inhibitor versus normalized response.

Based on the obtained IC50 (Table 2); the most potent three active compounds (naringenin, apigenin-7-*O*-glucoside, and acetoside) were selected to be tested for their anti-viral activity on SARS-CoV-2 (hCoV-19/Egypt/NRC-03/2020 (Accession Number on GSAID: EPI_ISL_430820). The results (Figure 5) indicated that only naringenin showed moderate anti-SARS-CoV-2 activity at non-cytotoxic concentrations in vitro with significant selectivity index (CC_50_/IC_50_ = 178.748/28.347 = 6.3). Naringenin (NAR) is a major flavanone in citrus fruits. Its combustion in experimental model reduces the production of proinflammatory cytokines [25] and regulate the production of IL-6 and TNF [26], that are increased in COVID-19. NAR also is known for its antiviral activity in SARS-CoV-2 infection [27]. It can reduce ACE2 expression [28], however this effect need further studies as reduction of ACE2 could result in greater inflammation [29]. Moreover, NAR was able to target the endo-lysosomal Two-Pore Channels (TPCs) in the Italian SARS-CoV-2 strain [30]. In addition, previous in silico studies demonstrated the ability of NAR to inhibit SARS-CoV-2 3CL^pro^ and consequently inhibit viral replication [31].

From clinical point of view, therapeutic potential and safety as well as pharmacokinetics and metabolism of NAR indicated its safety [32]. A dose of 600 mg in healthy volunteers resulted in a serum C_max_ of about 50 μM, without relevant toxicity [33]. In addition, the hydrophobic nature of NAR facilitates crossing of biological membrane and reach the cell in a suitable concentration [30].

**Figure 6:**
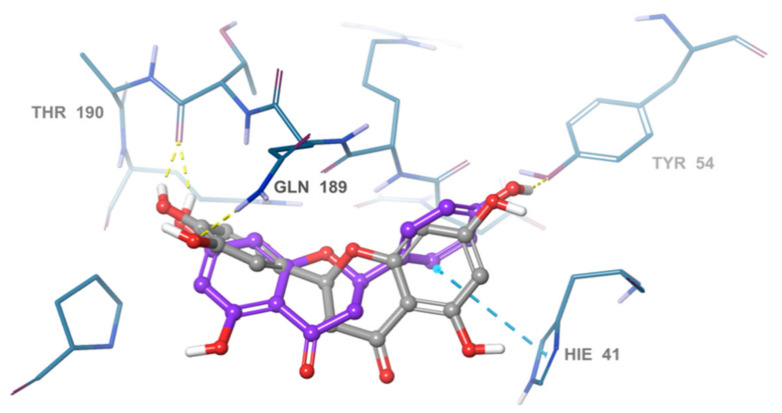
Overlay of eriodyctiol (grey), and naringenin (violet). in the SARS-COV-2 main protease active site.

In order to assess the structure-activity relationship of the most active compound naringenin, similar compounds were searched in the e-molecule site. (https://www.emolecules.com/, accessed on 25 March 2021) using the substructure search tool. Twelve closely related derivatives (Table S2, Figure S4) to naringenin were downloaded and docking of these compounds was carried out to the Mpro active site in comparison to naringenin. All compounds showed lower activity than naringenin as indicated by their lower score compared to that of naringenin, indicating that the virtual screening step, in the current research, effectively selected the most active hits among the available online databases. Eriodyctiol was the most active compound with a docking score of (−6.9), while (+)-eriodictyol-4′-methyl ether showed the lowest binding (−5.78) (Table S2). Alignment of the most active compound eriodyctiol (Figure 6) to that of naringenin in the active site showed reverse orientation and H-bonding for the hydroxyl groups in the B and A rings for both compounds. The 4′ position of eriodyctiol H-bonds to the glutamine 189 and threonine 190 residues, while naringenin binds to the same residues with its 7-OH position. On the other hand, eriodyctiol binds to tyrosine 54 in a similar way to that of the 4′ hydroxyl of naringenin, while it lacks any interaction with the most important residue (His 41) due to this orientation which could interpret its lower activity. Methoxylation of hydroxyl groups in naringenin molecule resulted in a marked decrease in activity. The 4′ methoxy derivative showed the lowest binding which confirms the importance of this hydroxyl in binding to residues in the active site (Figure 6). Adding one more hydroxyl group at 3′ position to naringenin resulted in a significant decrease in the binding score compared to that of naringenin as in eriodyctiol (−6.9). Adding one hydroxyl to the 3 positions as in aromadendrin resulted in a similar effect. The presence of two hydroxyl groups more than that of naringenin resulted in more decrease in binding as in dihydromorin and taxifolin. Again, methoxylating the hydroxyl group at 4′ of eriodyctiol resulted in a decrease in activity compared to the parent compound confirming the importance of this position. Finally, adding methoxy or methyl group to the 6- position of naringenin resulted in a decrease in the activity.

### 2.6. Pharmacophore Identification

**Figure 7:**
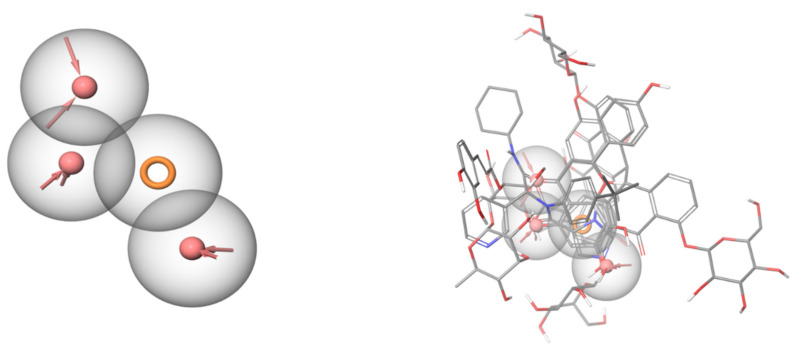
Common feature pharmacophore (left) generated using the active hits and the co-crystallized ligand. Red areas are acceptor features, and the orange ring is an aromatic ring feature. The active hits are aligned over each other (right) and mapped over the common feature pharmacophore. All the shown compounds map well to the features of the pharmacophore.

To understand the structure-activity relationship among this diverse groups of active in vitro hits, a common feature pharmacophore was generated. The pharmacophore model showed three common acceptor features and one common aromatic ring feature among all the active hits as well as the co-crystallized ligand (PDB ID: 6W63). All the active ligands were able to map well to the features of the pharmacophore model. Furthermore, naringenin was able to show the highest fitness value to the pharmacophore, which explains its high in vitro activity despite showing a relatively low docking score. This pharmacophore model gives some insight in the required feature for SARS-CoV-2 M^PRO^ inhibitors (Figure 7).

### 2.7. Drug Likeness

Drug likeness is a commonly used in drug discovery and development of orally bioavailable molecules. The parameters used are mainly based on Lipinski’s rule stating that; orally bioavailable drug should have a molecular mass less than 500 Dalton, high lipophilicity (expressed as LogP less than 5), less than 5 hydrogen bond donors, less than 10 hydrogen bond acceptors. A compound with two parameters outside the cutoff values indicates its proposed poor permeability or oral bioavailability [34,35]. Table 4 shows the calculated parameters for the most active compounds using Molinspiration chemoinformatics tool online. Naringenin and pentahydroxy benzophenones showed no violation of the drug likeness parameters indicating their good predicted oral bioavailability. However, acetoside and sennoside B showed a molecular weight of more than 500. In addition, acteoside, sennoside B and apigenin-7-glucoside violated the hydrogen bond donor/acceptor numbers.

TPSA is used as a good measure for prediction of drug transport properties. it is correlated efficiently with human intestinal absorption. naringenin and pentahydroxy benzophenone revealed a good TPSA value <140 Å indicating their possible intestinal permeability [36]. Again, the glycosides acetoside, sennoside B and apigenin −7 glucoside showed higher TPSA values indicating their poor permeability. Finally, % absorption of pentahydroxy benzophenone, naringenin and apigenin-7-glycoside were 50%, 68% and 78%, respectively indicating good absorption.

Deviation of FDA approved drugs from Lipinski’s rule of 5 is quite common, cardiac glycosides for example is one of the classes reported by Lipinski et al. 1997. In addition to vitamins, antibiotics and antifungals. The reason for these classes is attributed to having specific active transporters in the GIT or being a prodrug activated by hydrolyzing enzymes by the GIT microflora which in part can be applied for sennoside B and apigenin-7 glucoside. The overall results of naringenin regarding drug likeness parameters could be the reason for its promising inhibitory activity on cell based-virus replication due to its great permeability into cells.

## 3. Materials and Methods

### 3.1. Molecular Modeling

Commercially available compounds on the Zinc Database were collected and downloaded then converted to sdf format along with available in-house compounds. The sdf files were then prepared using Ligprep (2020, Schrödinger, LLC, New York, NY, USA). Standard ligand preparation settings were used without generation of tautomeric or ionization states. Only one prepared structure was generated for each ligand for rapid screening purposes. The SARS-CoV-2 M^PRO^ protein crystal structures co-crystallized with suitable inhibitors were downloaded from the PDB. Entry 6W63 containing a non-covalent inhibitor was used for non-covalent docking while entry 6Y2F containing a covalent inhibitor was used for covalent docking experiments. Both crystal structures were prepared using Schrödinger’s protein preparation wizard with default settings [37]. A docking grid was generated for 6W63 and used for non-covalent docking. Glide was used for docking where docking settings were set to default and standard precision was used [38]. For pose prediction of active compounds, extra precision docking was used. One output pose was generated for each ligand. 6Y2F was used for covalent docking with standard settings. Only one ligand, acetoside, was docked covalently using CovDock, where a Michael-acceptor type reaction was chosen [22]. A common feature pharmacophore was generated using Phase using standard settings [39].

### 3.2. Source of the Tested Isolates

Acteoside, iso-acteoside, sennoside B, and oleuropein were purchased from Biopurify Phytochemicals Ltd. (Chengdu, China). Thymoquinone was purchased from Sigma-Aldrich Inc. (St. Louis, Broadway, MO, USA). Meanwhile, all other tested compounds were obtained from in-house library of chemical compounds, 2,3′,4,5′,6-pentahydroxybenzophenone, maclurin-6-*O*-β-d-glucopyranoside, aromadendrin-8-*C*-β-d-glucopyranoside [40], 1,3,6,7-tetrahydroxy-8-prenylxanthone [41], kaempferol-7-*O*-β-d-glucoside, naringenin [42], apigenin-7-*O*-β-d-glucoside [43], sagitol C [44], 1-hydroxy-3,4-dihydronorharmane, butyrolactone I [45], terrenolide S [46], and ingenine C [47]

### 3.3. In Vitro Screening

In vitro screening of enzyme inhibition activities was done using 3CL Protease, Untagged (SARS-CoV-2) Assay Kit, Catalog #: 78042-1, BPS Bioscience, Inc., Allentown, PA, USA). According to manufacturer protocol a fluorescent substrate harboring the cleavage site (↓) of SARS-CoV-2 Mpro (Dabcyl-KTSAVLQ↓SGFRKM-E (Edans), 3CL protease (SARS-CoV-2 3CL Protease,), GenBank Accession No. YP_009725301, a.a. 1–306 (full length), expressed in *E. coli* expression system, MW 77.5 kDa., and buffer composed of 20 mM Tris, 100 mM NaCl, 1 mM EDTA, 1 mM DTT, pH 7.3 was used for the inhibition assay, GC376 a 3CL protease inhibitor, MW 507.5 Da was used as control. In the fluorescence resonance energy transfer (FRET)-based cleavage assay, the fluorescence signal of the Edans generated due to the 3CL Protease cleavage of the substrate was monitored at an emission wavelength of 460 nm with excitation at 360 nm, using a Flx800 fluorescence spectrophotometer (BioTek) [48]. Initially, 30 µL of diluted SARS-CoV-2 3CL protease at the final concentration of 15 ng was pipetted into a 96-well plate containing pre-pipetted 10 µL of test compounds. The mixture was incubated at room temperature for 30 min with slow shaking. Afterwards, the reaction was commenced by adding the substrate (10 µL) dissolved in the reaction buffer to 50 μL final volume, at concentration of 40 μM, incubated for 4 h at room temperature with slow shaking. The plates were sealed. Fluorescence intensity was measured in a microtiter plate-reading fluorimeter capable of excitation at a wavelength 360 nm and detection of emission at a wavelength 460 nm.

### 3.4. Drug Likeness

Drug likeness of the most active compounds were calculated using the molecular properties calculator of the free online Molinspiration Chemoinformatics tool (http://www.molinspiration.com, accessed on 25 March 2021) on 25-3-2021. The calculation is depending on the Lipinski’s rule of five [35] including Logarithm of partition coefficient between n-octanol and water (miLogP), molecular weight (MWt), number of hydrogen bond donors (nOHNH), number of hydrogen bond acceptors (nON). In addition, topological polar surface area (TPSA) [36] and the percentage absorption (%ABS) of the compounds were calculated by applying the following equation [49]: %ABS = 109 − [0.345XPSA].

### 3.5. Cytotoxicity Assay

To assess the half maximal cytotoxic concentration (CC_50_), stock solutions of the tested compounds were prepared in 10% DMSO in ddH_2_O and diluted further to the working solutions with DMEM. The cytotoxic activity of the tested compounds was tested in VERO-E6 cells by using the 3-(4, 5-dimethylthiazol-2-yl)-2, 5-diphenyltetrazolium bromide (MTT) method with minor modifications. Briefly, the cells were seeded in 96 well-plates (100 µL/well at a density of 3 × 10^5^ cells/mL) and incubated for 24 h at 37 °C in 5% CO_2_. After 24 h, cells were treated with various concentrations of the tested compounds in triplicates. 24 h later, the supernatant was discarded, and cell monolayers were washed with sterile 1× phosphate buffer saline (PBS) 3 times and MTT solution (20 µL of 5 mg/mL stock solution) was added to each well and incubated at 37 °C for 4 h followed by medium aspiration. In each well, the formed formazan crystals were dissolved with 200 µL of acidified isopropanol (0.04 M HCl in absolute isopropanol = 0.073 mL HCL in 50 mL isopropanol). Absorbance of formazan solutions was measured at λ max 540 nm with 620 nm as a reference wavelength using a multi-well plate reader. The percentage of cytotoxicity compared to the untreated cells was determined with the following equation.

The plot of % cytotoxicity versus sample concentration was used to calculate the concentration which exhibited 50% cytotoxicity (CC_50_):

$$\%\;\mathrm{cytotoxicity}=\frac{\left(\mathrm{absorbance}\;\mathrm{of}\;\mathrm{cells}\;\mathrm{without}\;\mathrm{treatment}\;-\;\mathrm{absorbance}\;\mathrm{of}\;\mathrm{cells}\;\mathrm{with}\;\mathrm{treatment}\right)\;\times \;100}{\mathrm{absorbance}\;\mathrm{of}\;\mathrm{cells}\;\mathrm{without}\;\mathrm{treatment}}$$

### 3.6. Inhibitory Concentration 50 (IC_50_) Determination

In 96-well tissue culture plates, 2.4 × 10^4^ Vero-E6 cells were distributed in each well and incubated overnight at a humidified 37 °C incubator under 5%CO_2_ condition. The cell monolayers were then washed once with 1× PBS and subjected to virus adsorption (hCoV-19/Egypt/NRC-03/2020 (Accession Number on GSAID: EPI_ISL_430820)) for 1 h at room temperature (RT). The cell monolayers were further overlaid with 100 μL of DMEM containing varying concentrations of the test compounds. Following incubation at 37 °C in 5% CO_2_ incubator for 72 h, the cells were fixed with 100 μL of 4% paraformaldehyde for 20 min and stained with 0.1% crystal violet in distilled water for 15 min at RT. The crystal violet dye was then dissolved using 100 μL absolute methanol per well and the optical density of the color is measured at 570 nm using Anthos Zenyth 200rt plate reader (Anthos Labtec Instruments, Heerhugowaard, The Netherlands). The IC_50_ of the tested compounds is that required to reduce the virus-induced cytopathic effect (CPE) by 50%, relative to the virus control.

## 4. Conclusions

The SARS-CoV-2 virus has caused an ongoing and rising pandemic of the disease COVID-19. Currently, there are still no medications with proven targeted activity towards this virus and many ongoing research projects worldwide aim to discover new potent medications for this disease. Our approach is to repurpose well studied natural products to become inhibitors of the virus’s main protease. This enzyme is a crucial component of the viral machinery since it processes the viral polypeptide into useful biomolecules for the virus. To accomplish our goal, we computationally screened more than 8000 natural products obtained from the Zinc Database and our in-house library. Eighteen of the screened compounds showed promising in silico results and were selected for further in vitro screening. Five of the tested compounds (naringenin, 2,3′,4,5′,6-pentahydroxybenzophenone, apigenin-7-*O*-glucoside, sennoside B, and acetoside) displayed high activity against the viral protein. Acteoside showed similar potency to the positive control GC376. The mechanism of inhibition and binding poses of each of the five compounds were determined in silico via extra precision molecular docking. Acteoside was found to bind to M^PRO^ non-covalently rather through a covalent mechanism. The most potent compounds were tested invitro on SARS-CoV-2 Egyptian strain where only naringenin showed moderate anti-SARS-CoV-2 activity at non-cytotoxic concentrations in vitro with significant selectivity index (CC_50_/IC_50_ = 178.748/28.347 = 6.3). A common feature pharmacophore model was generated to explain the requirements for enzyme inhibition by this diverse group of active ligands. The pharmacophore model showed three common acceptor features and one common aromatic ring feature among all the active hits as well as the co-crystallized ligand (PDB ID: 6W63). These results pave a path for future repurposing and development of natural products to aid in the fight against COVID-19. Moreover, we can conclude that NAR could be a promising drug candidate in treatment of COVID-19 infection. It could be consumed as a prophylactic or on the onset of SARS-CoV-2 infection to act as anti- SARS-CoV-2 through reduction of ACE2 expression, SARS-CoV-2 3CL^pro^ inhibition, and targeting TPCs. Clinical trials are needed to help understand the role of NAR consumption in humans during a viral infection, especially in COVID-19 infection.

## Figures and Tables

**Table 1:** The top hits of natural isolates with highest affinity to the viral protease (SARS-CoV-2 M^PRO^).

No.	Compound Name	Structure	Glide G-Score
**1**	Acteoside	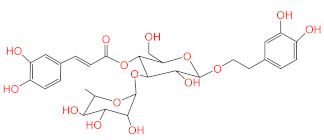	−10.127
**2**	Sennoside B	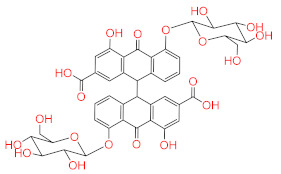	−9.009
**3**	2,3′,4,5′,6-Pentahydroxy benzophenone	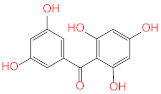	−8.341
**4**	Ochnaflavone-4′-methyl ether	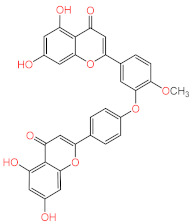	−8.310
**5**	Naringenin	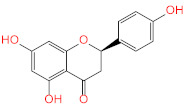	−7.824
**6**	Apigenin-7-*O*-β-d-glucoside	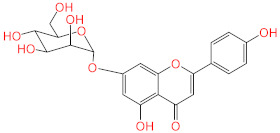	−7.562
**7**	Sagitol C	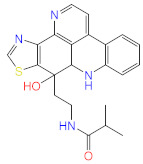	−7.505
**8**	Maclurin-6-*O*-β-d-glucopyranoside (Rhodanthenone)	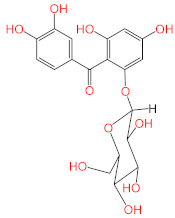	−7.441
**15**	Iso-Acteoside	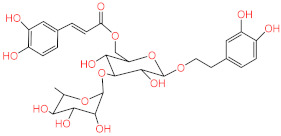	−7.724
**16**	Kaempferol-7-*O*-β-d-glucoside	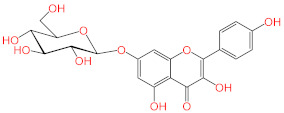	−7.206
**17**	Thymoquinon	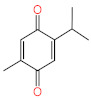	−6.693
**18**	Oleuropein	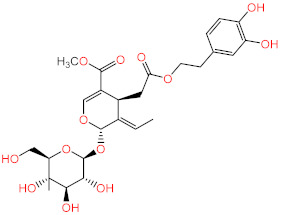	−5.509

**Table 2:** IC_50_ of most active compounds compared to GC376 (Standard 3CL Protease enzyme inhibitor) on SARS COV-2 viral main protease.

Compound Name	IC_50_ (nM)
2,3′,4,5′,6-Pentahydroxybenzophenone	102
Sennoside B	104
Naringenin	92
Apigenin-7-*O*-β-d-glucoside	74
Acteoside	43
GC376 (positive control)	44

**Table 3:** Interaction of natural compound inhibitors with active site residues of SARS-CoV-2 M^PRO^.

Compound Name	Hydrogen Bond	Pi-Pi Stacking	Van der Waal Interactions	XP GScore
Acteoside	Cys44, Met49, Asn142, Hie164, Glu166, Thr190		Pro168, Glu189, Thr25, Hie41, Cys145, Met49	−12.341
Apigenin-7-*O*-β-d-glucoside	Thr26, Met49, Glu166, Gln189, Thr190	Hie41	Met49, Thr25, Hie41	−10.834
Sennoside B	Thr190, Glu166, Asn142, Cys44		Pro168, Gln189, Met49	−10.336
2,3′,4,5′,6-pentahydroxybenzophenone	Hie164, Glu166, Arg188, Tyr54		Hie41, Met49, Met165, Pro168	−8.105
Naringenin	Tyr54 Thr190		Hie41, Met49, Met165, Pro168	−7.083

**Table 4:** Predicted molecular properties of the most active SARS-COV-2 Mpro inhibitors.

Compound	miLogP	TPSA	natoms	MW	nON	nOHNH	nrotb	% ABS
Acetoside	0.4	245.2	44	624.5	15	9	11	24.4%
Sennoside B	0.8	347.9	62	862.7	20	12	9	-
Naringenin	2.1	86.9	20	272.2	5	3	1	78.9%
Apigenin-7-glucoside	0.6	170.0	31	432.3	10	6	4	50.35
2,3′,4,5′,6-Pentahydroxybenzophenone	1.7	118.2	19	262.2	6	5	2	68.2%

MiLogP: Logarithm of partition coefficient between n-octanol and water. TPSA: Topological polar surface area. Non: Number of hydrogen bond acceptor. nOHNH: Number of hydrogen bond donors. Nrotb: Number of rotable bonds. MW: molecular weight. Nviolations: number of violations.

## Data Availability

Data is contained within the article and Supplementary Materials.

## Supplementary Materials

The following are available online at https://www.mdpi.com/1424-8247/14/3/213/s1, Figure S1. Superposition of the co-crystallized ligand (pink) and the redocked pose (green) with very small deviation and an RMSD value of 1.56 Å, Figure S2. 2D Interaction diagrams of acteoside, apigenin-7-O-glucoside, sennoside B, 2,3’,4,5’,6-pentahydroxy benzophenone, and naringenin, respectively inside the Mpro active site in the crystal form, Figure S3. 3D Interaction diagrams of acteoside, apigenin, sennoside B, 2,3’,4,5’,6-pentahydroxy benzophenone, and naringenin, respectively, Figure S4. Overlay of naringenin analogues (grey) with naringenin (violet) in MPro active site, Table S1. The inactive natural isolates with high affinity to the viral protease (SARS-CoV-2 MPRO), Table S2. Similar compounds to naringenin that searched for their activity against the viral protease (SARS-CoV-2 M^PRO^.
