# Supplementary material for: Repurposing of Some Natural Product Isolates as SARS-COV-2 Main Protease Inhibitors via In Vitro Cell Free and Cell-Based Antiviral Assessments and Molecular Modeling Approaches
Source: Pharmaceuticals (Basel). 2021 Mar 4;14(3):213. doi: 10.3390/ph14030213 (PMC8001104; doi:10.3390/ph14030213)
Supplement: Supplementary file 1 [file pharmaceuticals-14-00213-s001.pdf]

Repurposing of Some Natural Product isolates as SARS-COV-2 Main Protease Inhibitors via In vitro Cell Free and cell based anti-viral Assessments and Molecular Modeling Approaches

Hossam M. Abdallah[1,2,*,#], Ali M. El-Halawany [2,#], Alaa Sirwi[1] , Amr M. El-Araby[3], Gamal A. Mohamed[1,4], Sabrin R. M. Ibrahim[5,6], Abdulrahman E. Koshak[1] , Hani Z. Asfour[7], Zuhier A. Awan[8] and Mahmoud A. Elfaky[1]

Supplementary Data

Table S1. The inactive natural isolates with high affinity to the viral protease (SARS-CoV-2 M[PRO]).

No.	Compound Name	Structure	Glide G-Score
9	1-Hydroxy-3,4-dihydronorharmane	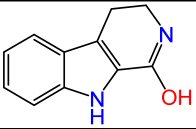	-7.43
10	Aromadendrin-8-C-β-D-glucopyranoside	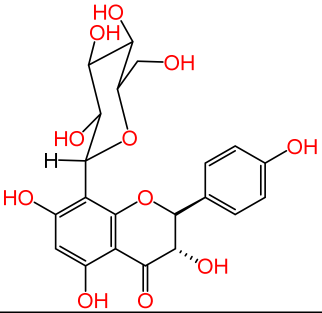	-7.394
11	Butyrolactone I	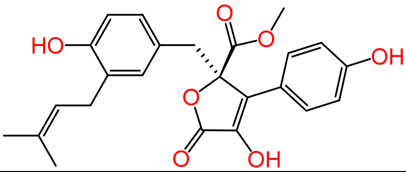	-7.316
12	Terrenolide S	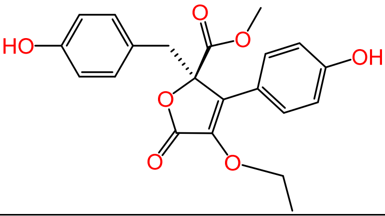	-7.304
13	Ingenine C	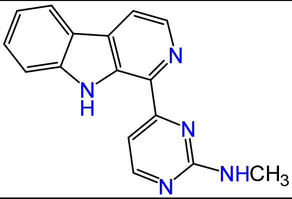	-7.286

14	1,3,6,7-Tetrahydroxy-8-prenylxanthone	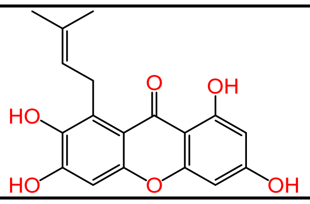	-7.284

Table S2. Similar compounds to naringenin that searched for their activity against the viral protease (SARS-CoV-2 M^{PRO}).

No	Compound Name	Structure	G-score
1	(+)-Eriodictyol	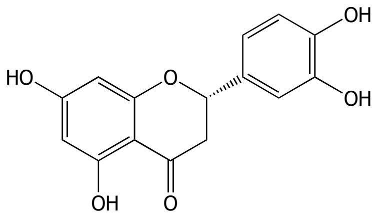	-6.933
2	(+)Naringenin-5-methyl ether	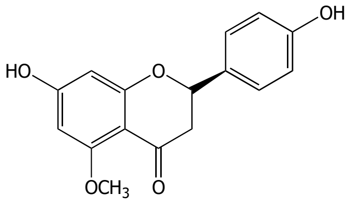	-6.854
3	Naringenin-6`-methyl ether	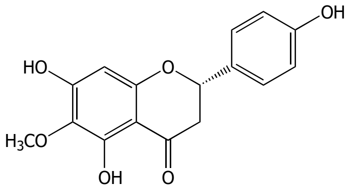	-6.816
4	(-) Aromadendrin	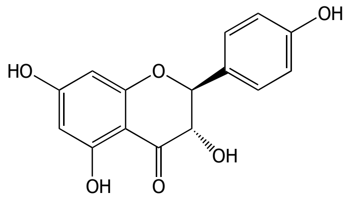	-6.754
5	(+)6,8-dimethyl Naringenin	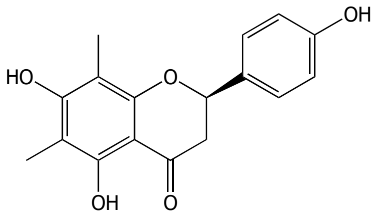	-6.719

6	(+)Naringenin-3`-methoxy	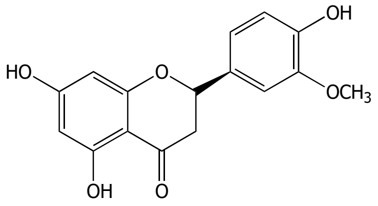	-6.675
7	Dihydromorin	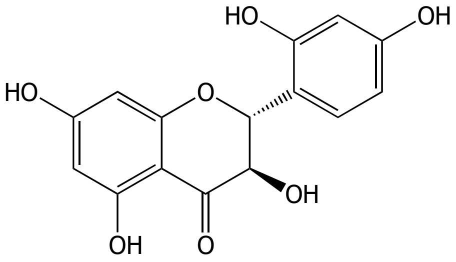	-6.618
8	(+) Naringenin-7-methyl ether	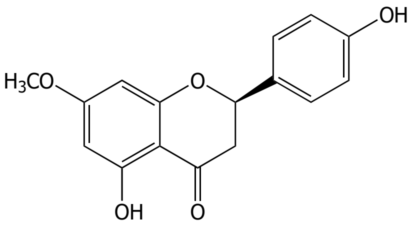	-6.617
9	(+)Naringenin-7,4`-dimethyl ether	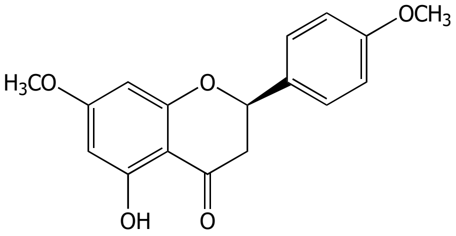	-6.543
10	Naringenin-4`-methyl ether	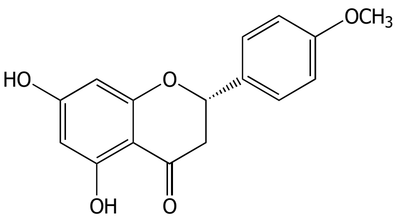	-6.511
11	(-)-Taxifolin	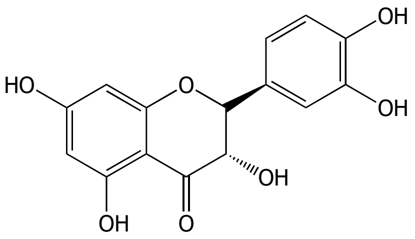	-6.494

12	(+)Eriodictyol-4`-methyl ether	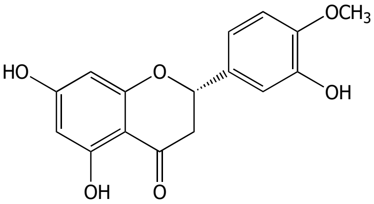	-5.783

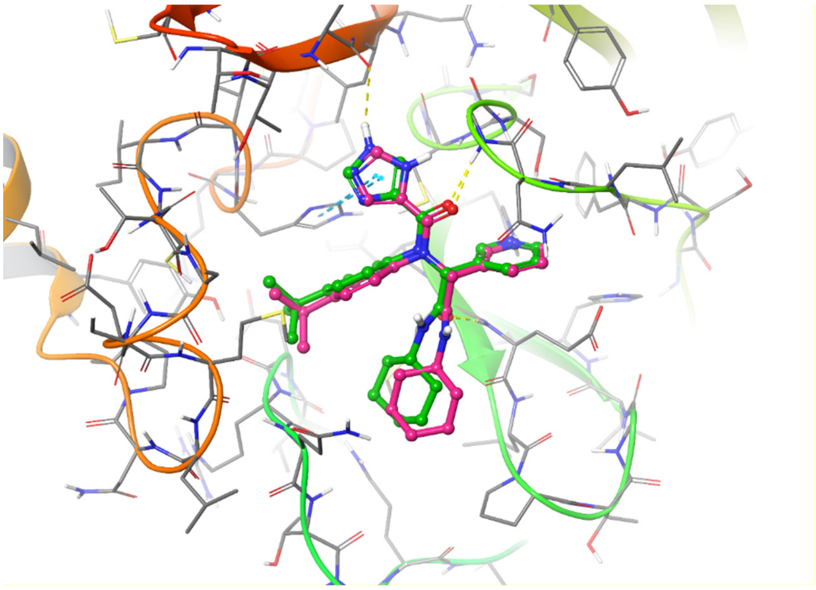

Figure S1. Superposition of the co-crystallized ligand (pink) and the redocked pose (green) with very small deviation and an RMSD value of 1.56 Å.

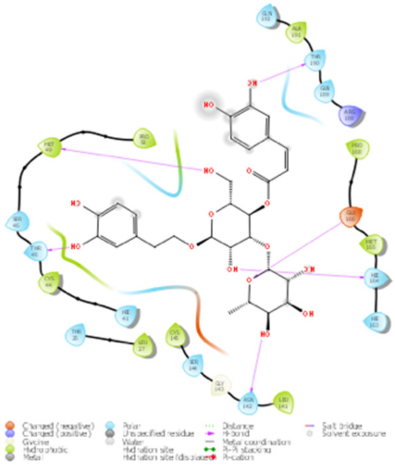

Acteoside

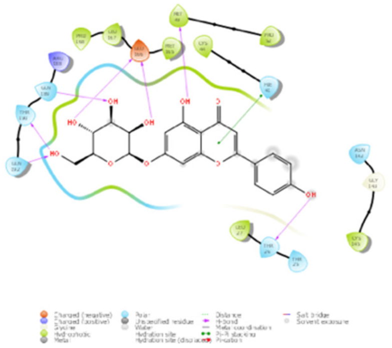

Apigenin-7-O-β-D-glucoside

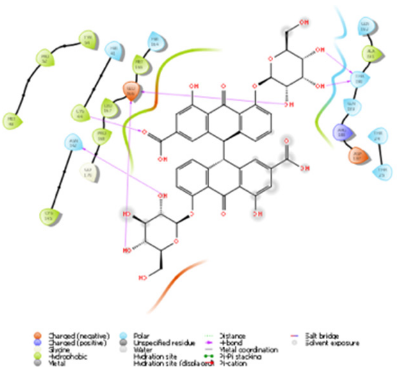

Sennoside B

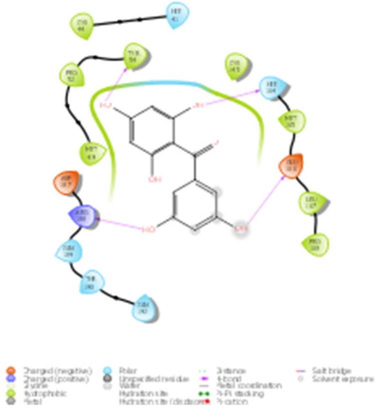

2,3',4,5',6-Pentahydroxy benzophenone

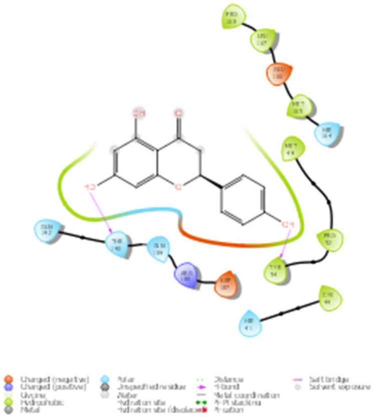

Naringenin

Figure S2. 2D Interaction diagrams of acteoside, apigenin-7-O-glucoside, sennoside B, 2,3',4,5',6-pentahydroxy benzophenone, and naringenin, respectively inside the Mpro active site in the crystal form.

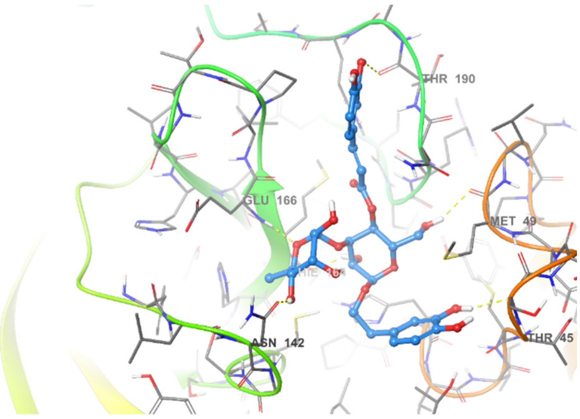

Acteoside

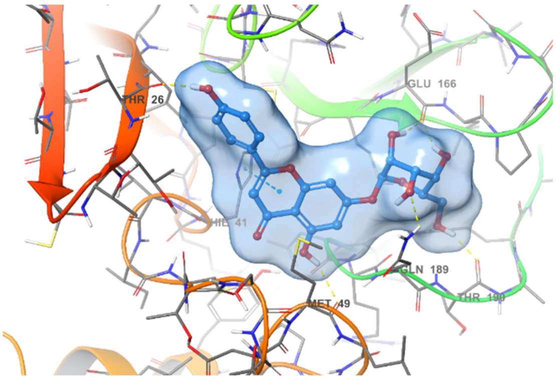

Apigenin-7-O-β-D-glucoside

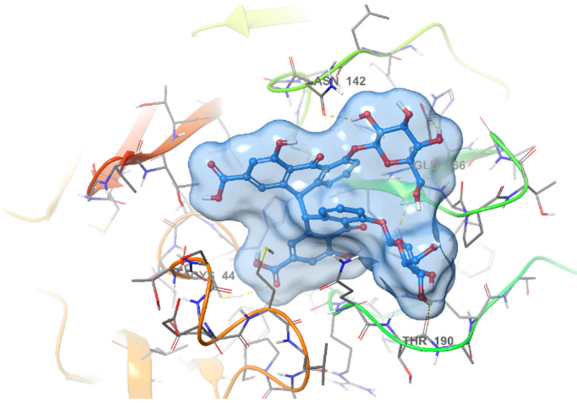

Sennoside B

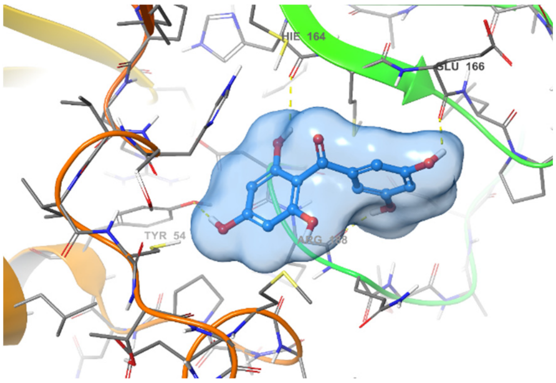

2,3',4,5',6-Pentahydroxy benzophenone

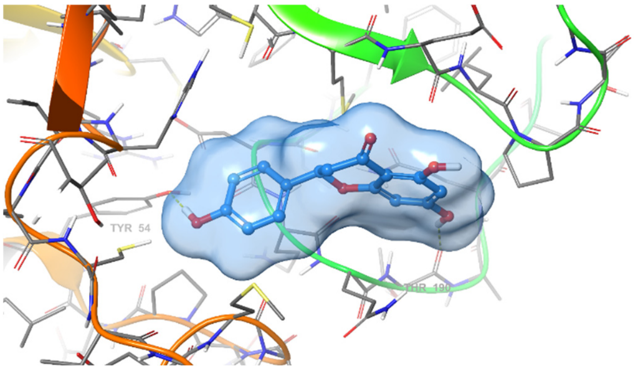

Naringenin

Figure S3. 3D Interaction diagrams of acteoside, apigenin, sennoside B, 2,3',4,5',6-pentahydroxy benzophenone, and naringenin, respectively.

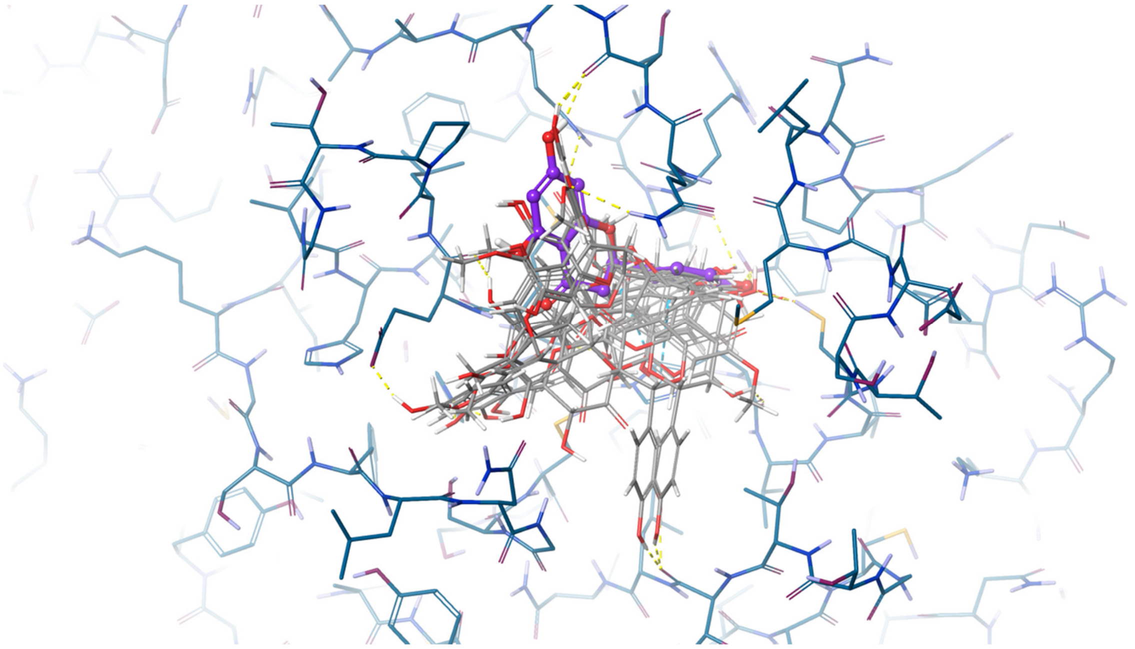

Figure S4. Overlay of naringenin analogues (grey) with naringenin (violet) in MPro active site
